# Effect of Chum Salmon Egg Lectin on Tight Junctions in Caco-2 Cell Monolayers

**DOI:** 10.3390/molecules20058094

**Published:** 2015-05-05

**Authors:** Ryo Nemoto, Shintaro Yamamoto, Tomohisa Ogawa, Ryno Naude, Koji Muramoto

**Affiliations:** 1 Graduate School of Life Sciences, Tohoku University, Katahira 2-1-1, Aoba-ku, Sendai 980-8577, Japan; E-Mails: timtam200g@gmail.com (R.N.); shin0708taro@gmail.com (S.Y.); ogawa@biochem.tohoku.ac.jp (T.O.); 2 Department of Biochemistry and Microbiology, Nelson Mandel Metropolitan University, Port Elizabeth 6031, South Africa; E-Mail: ryno.naude@nmmu.ac.za

**Keywords:** Caco-2 cell, intestinal transport, lectin, tight junction, transepithelial transport

## Abstract

The effect of a chum salmon egg lectin (CSL3) on tight junction (TJ) of Caco-2 cell monolayers was investigated. The lectin opened TJ as indicated by the decrease of the transepithelial electrical resistance (TER) value and the increase of the permeation of lucifer yellow, which is transported via the TJ-mediated paracellular pathway. The effects of CSL3 were inhibited by the addition of 10 mM L-rhamnose or D-galactose which were specific sugars for CSL3. The lectin increased the intracellular Ca^2+^ of Caco-2 cell monolayers, that could be inhibited by the addition of L-rhamnose. The fluorescence immunostaining of β-actin in Caco-2 cell monolayers revealed that the cytoskeleton was changed by the CSL3 treatment, suggesting that CSL3 depolymerized β-actin to cause reversible TJ structural and functional disruption. Although Japanese jack bean lectin and wheat germ lectin showed similar effects in the decrease of the TER values and the increase of the intracellular Ca^2+^, they could not be inhibited by the same concentrations of simple sugars, such as D-glucose and *N*-acetyl-D-glucosamine.

## 1. Introduction

Lectins are a group of non-catalytic sugar-binding proteins that are widely distributed in varying amounts in most common foods [1,2]. Such lectins as the legume type have tolerance against heating, acidic pH and proteolytic digestion; the digestive tract is therefore constantly exposed to the biologically active lectins contained in fresh and processed foods [3–5]. Since the epithelial surface of the intestines is extensively glycosylated, lectins interact with this surface and can induce physiological effects on humans and other animals, particularly when consumed in large quantities. It is known that a high dose of uncooked or partially cooked kidney beans causes food poisoning [6]. The lectins contained in foods are therefore frequently regarded as anti-nutritional factors, although most of these adverse effects are limited to the legume lectins. Since the dietary intake of lectins is generally low, their activities have no measurable negative effects on their nutritional value. Moreover, in some cases, small amounts of lectins may have beneficial effects on a biological system, such as promoting gut regrowth after total parenteral nutrition, use as an oral vaccine adjuvant, and use in anti-cancer therapy [3,6].

The absorption of nutrients and food factors across the intestinal epithelium occurs because of one or more different transport pathways, such as passive paracellular transport, passive transcellular transport, and carrier-mediated transport [7]. The human colon adenocarcinoma cell line, Caco-2, has been used as an *in vitro* model of the human small intestinal epithelial system. Completely differentiated and polarized Caco-2 cell monolayers spontaneously exhibit various enterocyte characteristics, including the expression of brush border enzymes, nutrient transporters, and the formation of intercellular tight junctions (TJs) [8,9]. Dietary substances and drugs have been found to affect intestinal absorption in model Caco-2 cell monolayers by using fluorescent markers, including lucifer yellow (LY) for the paracellular pathway [10,11]. The transepithelial electrical resistance (TER) value across a Caco-2 cell monolayer reflects any effects on the TJ-mediated paracellular pathway. Various dietary components have been shown to regulate epithelial permeability by altering the expression and localization of TJ proteins [7,12]. We have shown that lectins contained in foodstuff had varying modulating effects on the transport system of human intestinal Caco-2 cell monolayers [13,14,15]. For example, the transport of LY was increased by wheat germ lectin (WGA), peanut lectin (PNA), white mushroom lectin (ABA), *Aspergillus oryzae* lectin (AOL), and chum salmon egg lectin (CSL3).

Among the lectins tested, CSL3 showed the most prominent effects toward various transport systems [14]. CSL3 is an l-rhamnose (Rha) binding lectin isolated from chum salmon (*Oncorhynchus keta*) eggs, which are known as “ikura” and widely consumed, not only in Japan, but also in other countries, in the form of caviar. CSL3 is a homodimer of two 20 kDa subunits with a dumbbell-like shape overall, in which the N- and C-terminal domains of different subunits form lobe structures connected with flexible linker peptides [16,17].

The present study investigated the effects of a chum salmon egg lectin, CSL3, on TJ of Caco-2 cell monolayers in more detail, to gain insight concerning the function of food lectins. For comparison, the effects of Japanese jack bean lectin (CGA) and wheat germ lectin (WGA) were also examined.

## 2. Results and Discussion

### 2.1. Effect of Lectins on the TER Values

**Figure 1:**
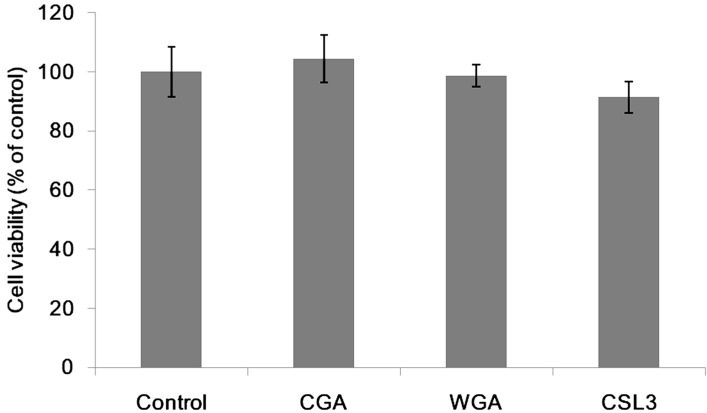
Cytotoxicity of lectins on Caco-2 cells. Cytotoxicity was measured after incubating with lectins (200 µg/mL) for 6 h. Results are shown as a percentage of control (without lectins) values, and are the means ± SD of three different determinations.

Differentiated Caco-2 cells were incubated with lectins for 6 h to examine their cytotoxic activity against the cells (Figure 1).

**Figure 2:**
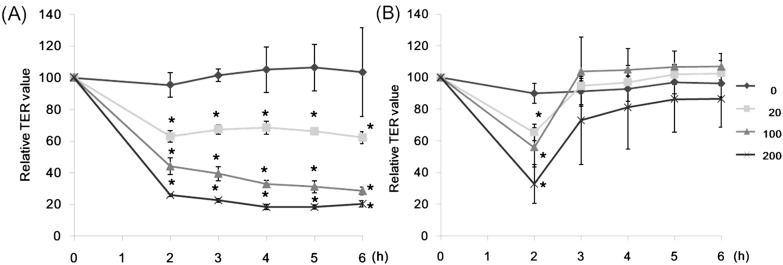
Effects of CSL3 on TER values of Caco-2 cell monolayers. (**A**) The cell monolayers were incubated with CSL3 (20–200 µg/mL) for 6 h. TER values were measured periodically during incubation. (**B**) The cell monolayers were incubated with CSL3 (20–200 µg/mL). After incubation with CSL3 for 2 h, 10 mM l-rhamnose were added to the apical side. Results are shown as a percentage of the control (without lectins) values, and are the means ± SD of three different determinations. * *p* < 0.05 compared with the control value at 2 h.

**Figure 3:**
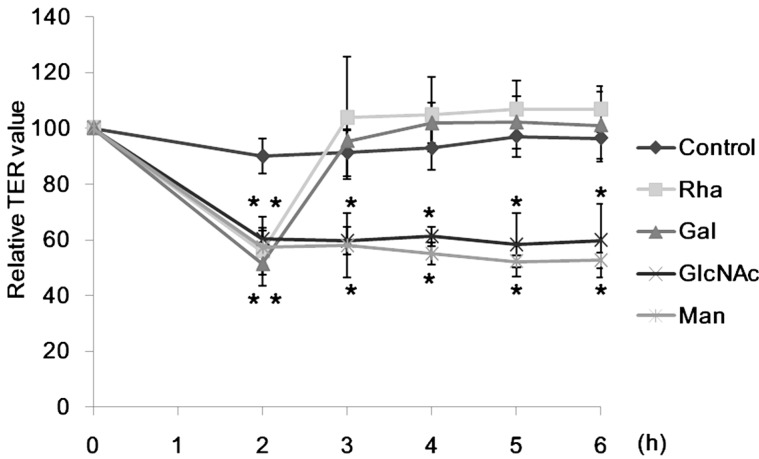
Effects of sugars on TER values after being decreased by CSL3. The Caco-2 cell monolayers were incubated with CSL3 (200 µg/mL) for 6 h. TER values were measured periodically during incubation. After incubation with CSL3 for 2 h, 10 mM sugar was added to the apical side. Results are shown as a percentage of the control (without lectins) values, and are the means ± SD of three different determinations. * *p* < 0.05 compared with the control value at 2 h.

None of lectins showed apparent cytotoxicity even at the highest concentration (200 µg/mL) tested, though CSL3 caused apoptosis of the cells when incubated for 24 h [17]. The TER value can be an indicator of the tightness of intercellular junctions; a decreased TER value indicates an increase of the paracellular transport, or *vice versa* [11]. Caco-2 cell monolayers were incubated with various concentrations of CSL3 for 2–6 h and their TER values were measured (Figure 2A). CSL3 decreased the TER values significantly after 2-h incubation. This effect continued until 6 h of incubation and the TER values decreased in a concentration dependent manner. By the addition of 10 mM l-rhamnose (Rha), the TER values, after being decreased by CSL3, quickly increased to the control level (Figure 2B). A similar effect was also found in the presence of d-galactose (Gal), but not with *N*-acetyl-D-glucosamine (GlcNAc) or D-mannose (Man) (Figure 3). The addition of Rha or Gal without lectins did not affect TER values. CSL3 has a specific binding affinity for Rha and Gal, and a much stronger binding affinity (*Kd* = 2.6 × 10^−5^ M) for globotriaosylceramide β(Gb3; Galα1-4Galβ1-4Galβ1-Cer) which is located in lipid rafts on a cell surface [16].

**Figure 4:**
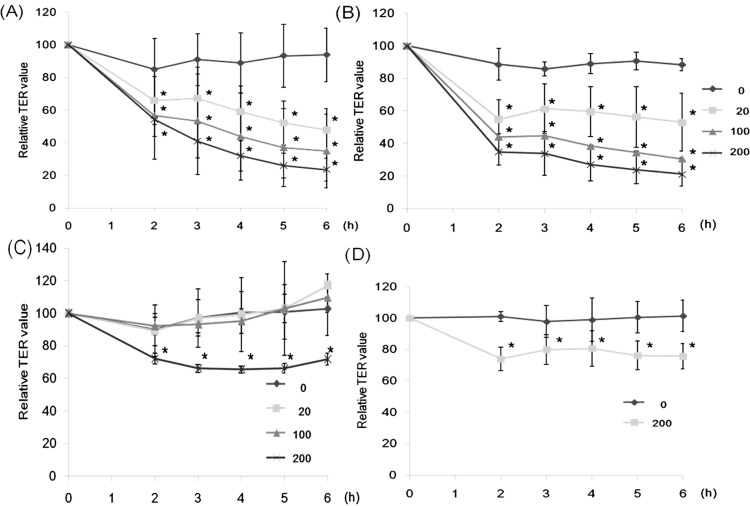
Effects of CGA and WGA on TER values of Caco-2 cell monolayers. The cell monolayers were incubated with CGA (20–200 µg/mL) (**A**) or WGA (20–200 µg/mL) (**C**) for 6 h. TER values were measured periodically during incubation. After incubation with CGA or WGA for 2 h, 10 mM D-glucose (**B**) and 10 mM *N*-acetyl-d-glucosamine (**D**) were added to the apical side, respectively. Results are shown as a percentage of the control (without lectin) values, and are the means ± SD of three different determinations. * *p* < 0.05 compared with the control values at 2 h.

Although CGA also decreased the TER values, its specific sugar, D-glucose (Glc), failed to increase the values at 10 mM (Figure 4B). WGA showed only a weak effect on the decrease of the TER values even at 200 µg/mL (Figure 4C). The decreased values could not be increased by the addition of its specific sugar, GlcNAc at 10 mM (Figure 4D). These results suggest that CSL3 interacts with Caco-2 cells specifically to affect TJ in a different manner as compared to CGA or WGA.

**Figure 5:**
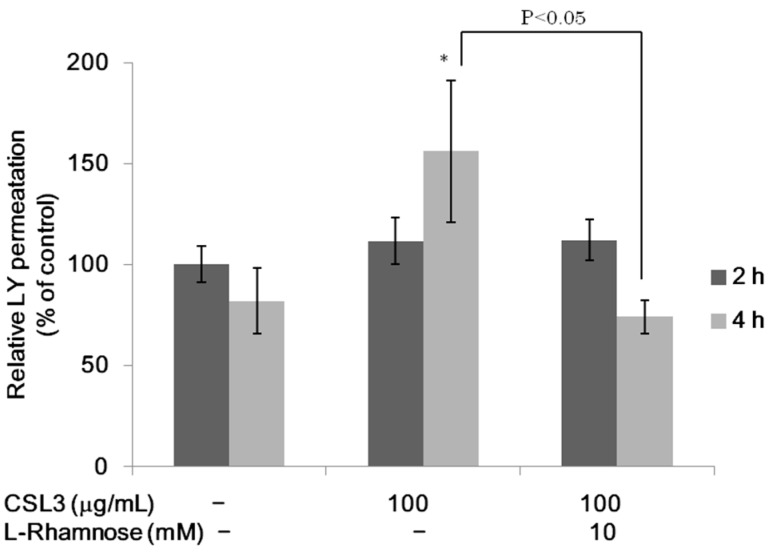
Effect of CSL3 on LY transport across Caco-2 cell monolayers. LY in the basolateral side was measured after incubating with CSL3 for 2–4 h. After incubation with CSL3 for 2 h, 10 mM l-rhamnose was added to the apical side. Results are shown as a percentage of the control (without lectins) value, and are the means ± SD of three different determinations. * *p* < 0.05 compared with the control value at 2 h.

### 2.2. Effect of CSL3 on LY Transport

LY is transported via a paracellular pathway and is known as an indicator of TJ [11]. Although CSL3 did not increase the permeation of LY significantly at 2 h, it increased significantly at 4 h (Figure 5). This effect at 4 h was negated by the addition of 10 mM Rha. This result indicates that the effect of CSL3 is closely related to its sugar binding activity.

### 2.3. Effect of CSL3 on the Intracellular Ca^2+^

**Figure 6:**
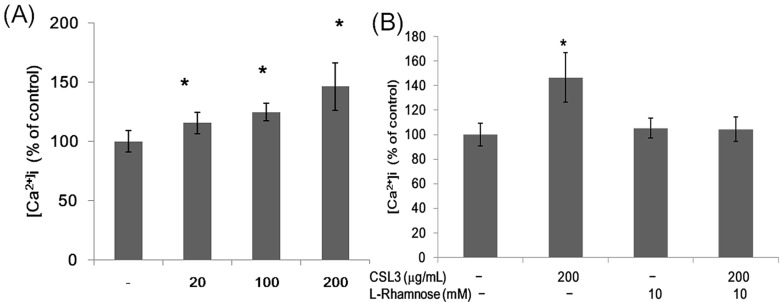
Effect of CSL3 on intracellular Ca^2+^ ([Ca^2+^]_i_) of Caco-2 cell monolayers. (**A**) The cells were incubated with CSL3 for 2 h and the changes in intracellular Ca^2+^ levels were measured by using a fluorescent Ca^2+^ probe. (**B**) The cells were treated with CSL3 for 2 h. After the addition of l-rhamnose, the cells were incubated for another 1 h, and the changes in the intracellular Ca^2+^ levels were measured by using a fluorescent Ca^2+^ probe. The results are shown as a percentage of the control (without lectins) values, and are the means ± SD of six different determinations. * *p* < 0.05 compared with control value.

CSL3 increased the intracellular Ca^2+^ significantly after a 2-h incubation in a dose dependent manner (Figure 6A). This effect was negated by the addition of Rha (Figure 6B). It has been reported that TJ is opened by changing the structure of cytoskeleton due to the increase of intracellular Ca^2+^ [18]. This study also showed that the intracellular Ca^2+^ of Caco-2 cell monolayers increased by the treatment with CSL3. CGA and WGA also increased the intracellular Ca^2+^, however, Glc and GlcNAc did not negate the effect at the concentrations used in this study (data not shown).

### 2.4. Effect of CSL3 on β-Actin

**Figure 7:**
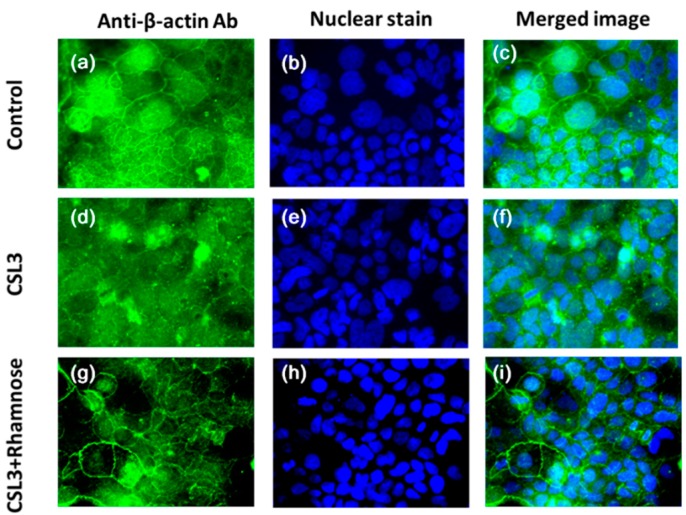
Effect of CSL3 on the distribution of β-actin in Caco-2 cell monolayers. The cells were incubated in HBSS for 2 h in the absence (**a**, **b**, **c**) or presence of CSL3 (100 µg/mL) (**d**, **e**, **f**). After incubation with CSL3, the cells were incubated with 10 mM l-rhamnose for another 1 h (**g**, **h**, **i**). The fluorescence images were examined by a fluorescence microscope.

The fluorescence immunostaining of Caco-2 cells is shown in Figure 7. In the control cells, β-actin formed a homogeneous continuum stained between the cells at the contact sides. CSL3 disturbed the clear staining of β-actin, while the addition of Rha restored the β-actin localization to the same level as the control.

Actin participates in many important cellular processes, including cell motility, cell division and cytokinesis, and the establishment and maintenance of cell junctions and cell shape. Many of these processes are mediated by biochemical intricate interactions of actin with cellular membranes. The β-actin coexists in most cell types as a component of the cytoskeleton, and as mediators of internal cell motility. The result suggests that CSL3 increased the intracellular Ca^2+^ to depolymerize β-actin in the cytoskeleton, resulting in reversible TJ structural and functional disruption. Although many more studies are required to reveal the complete mechanism underlying CSL3-induced TJ opening, there is some reports containing related information. Capsaicin, which is an active component of chili peppers, induces Ca^2+^ influx and cofilin dephosphorylatin in Caco-2 cells, and thereby causes TJ opening [19]. Cofilin is one of the actin-depolymerizing factors that directly regulate actin filament dynamics.

### 2.5. Effect of CSL3 on Claudin-1 Expression

**Figure 8:**
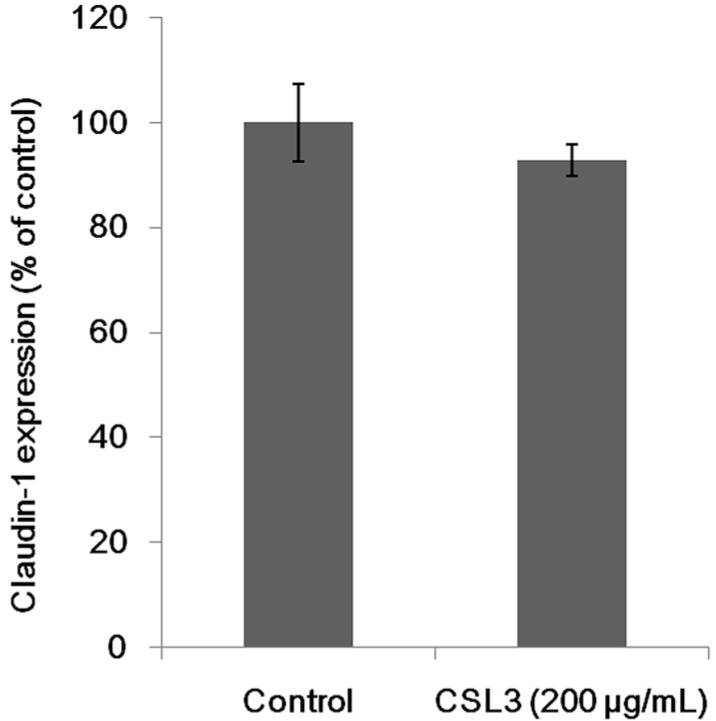
Effect of CSL3 on the expression level of claudin-1.The cells were incubated in HBSS for 2 h in the absence or presence of CSL3 (200 µg/mL). The results are shown as a percentage of the control (without lectins) values, and are the means ± SD of six different determinations. * *p* < 0.05 compared with control value.

The expression of claudin-1, a structural protein of TJ, was analyzed by Western blotting (Figure 8). CSL3 did not affect the claudin-1 expression. TJ is formed on an actin filament which is formed by polymerizing G-actin molecules. Therefore, it is possible that TJ is modulated by depolymerization of F-actin. Intracellular Ca^2+^ activates gelsolin, an actin filament-severing protein, and inactivates cofilin, an actin depolymerizing protein [19]. Moreover, it is known that many factors change the expression and/or localization of TJ structural proteins [20]. In addition, several plant lectins, such as soybean lectin (SBA), WGA and CGA, regulated the expression of 19 proteins including chaperon proteins and cytoskeleton related proteins, in Caco-2 cells after the treatment with lectins for 24 h [21]. CSL3 did not change the expression level of claudin-1, a TJ structural protein, under the conditions described herein. It is more likely that the lectin changes the localization of the cytoskeleton associated proteins.

### 2.6. Effect of CSL3 on TER Values in Gb3-Defective Caco-2 Cell Monolayers

**Figure 9:**
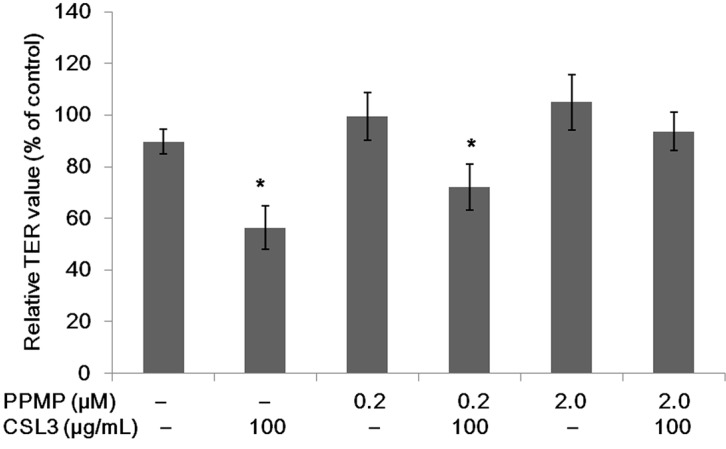
Effect of CSL3 on TER values of Caco-2 cell monolayers treated with PPMP to suppress the Gb3 expression. The TER values of the monolayers were measured after incubating with CSL3 (100 µg/mL) for 2 h. Results are shown as the percentage of the control (without PPMP and CSL3) values, and are the means ± SD of three different determinations. * *p* < 0.05 compared with the control value.

The Caco-2 cell monolayers treated with PPMP, an inhibitor of glucosylceramide synthesis, were incubated with CSL3 for 2 h, and their TER values were measured (Figure 9). The PPMP treatment weakened the effect of CSL3 in the decrease of the TER value of Caco-2 cell monolayers. Therefore, CSL3 did not show any effect on the cell monolayers treated with 2 μM PPMP, indicating that the lectin interacted with Gb3 to open TJ.

Food components, such as fatty acids, oligo- and polysaccharides, amino acids, peptides, proteins, and flavonoids have been shown to modulate TJ permeability by intracellular signaling [7,12]. Several polyphenols, including kaempferol and theaflavins, enhanced TJ integrity by affecting cytoskeletal association, the expression of TJ proteins, or microdomains in the membranes of Caco-2 cell monolayers [22,23]. On the other hand, capsaicin and chitosan opened TJs by inducing intracellular redistribution of the TJ proteins, claudin-1 and claudin-4, respectively [19,24]. The present study suggests that CSL3 modulates TJ permeability by a unique manner differing from those of known effectors, though further study is required to reveal the exact mechanism.

## 3. Experimental Section

### 3.1. Materials

CSL3, CGA and WGA were isolated as described previously [14]. The human colon adenocarcinoma cell line, Caco-2, was obtained from the American Type Culture Collection (Rockville, MD, USA). Dulbecco’s modified Eagle’s medium (DMEM), non-essential amino acids (NEAAs), penicillin-streptomycin (10,000 units/mL and 10 mg/mL in 0.9% NaCl), Lucifer yellow dilithium salt (LY), dl-*threo*-1-phenyl-2-palmitoylamino-3-morpholino-1-propanol (PPMP), anti-β-actin IgG antibody, anti-glyceraldehyde 3-phosphate dehydrogenase (GAPDH), and goat anti-rabbit IgG were purchased from Sigma (St. Louis, MO, USA). Fetal bovine serum (FBS) was obtained from Termo Science Hyclone (Waltham, MA, USA). Plastic dishes, 6-well dishes and 96-well microplates were from Becton Dickinson and Company (Franklin Lakes, NJ, USA). Transwell inserts with a 0.40-μm polycarbonate membrane of 6.5 mm in diameter were purchased from Corning Costar (Corning, NY, USA). WST-1 assay kit and calcium assay kit were from Dojindo Laboratories (Kumamoto, Japan). Alexa Fluor 488 conjugated anti-rabbit IgG antibody was from Life Technologies (Carlsbad, CA, USA). Horseradish peroxidase (HRP)-conjugated goat anti-rabbit IgG was from Jackson Immuno Research Laboratories (West Grove, PA, USA). All other chemicals used in this study were of analytical grade.

### 3.2. Cell Culture and TER Measurement

Caco-2 cells (passage number, 7–35) were cultured in DMEM with 10% (*v*/*v*) FBS, penicillin-streptomycin (50 IU/mL and 50 μg/mL, respectively), and 1% (*v*/*v*) NEAA. The cells were maintained at 37 °C in a humidified atmosphere of 5% CO_2_ in air. The cells were sub-cultured at 70%–80% confluency.

For cytotoxicity assay, Caco-2 cell monolayers were grown on 96-well plates at a density of 1.0 × 10^5^ cells/cm^2^, and maintained for 18–21 days with replacing culture medium every 2–3 days. Lectins were dissolved in Hank’s balanced salt solution (HBSS). The cell monolayers were gently rinsed twice with HBSS, and treated with lectins (200 µg/mL) for 6 h. After the treatment, the cytotoxicity was measured by using a WST-1 assay kit. The WST-1 reagent was incubated with the cells for another 2 h, and then the absorbance at 450 nm was measured by a microtiter plate reader (Bio Rad, Tokyo, Japan).

For transport experiments, Caco-2 cell monolayers were prepared by seeding on Transwell inserts in 24-well plates at a density of 1.0 × 10^5^ cells/cm^2^. The apical and basolateral compartments contained 0.1 and 0.6 mL of culture medium, respectively. The cell monolayers were maintained for 18–21 days and culture medium was replaced every 2–3 days. The integrity of the cell monolayers was evaluated by measuring the transepithelial electrical resistance (TER) value with a MillicelL-ERS equipment (Millipore, MA, USA). The cell monolayers showing TER values of more than 500 Ω/cm^2^ were used for the experiments.

For intracellular Ca^2+^ measurement, Caco-2 cell monolayers were grown on glass-bottom 96-well plates at a density of 1.0 × 10^5^ cells/cm^2^. The cell monolayers were maintained for 18–21 days with replacing culture medium every 2–3 days.

For fluorescence immunostaining, Caco-2 cell monolayers were grown on 30-mm diameter plates at a density of 1 × 10^5^ cells/well. The cell monolayers were maintained for 18–21 days and culture medium was replaced every 2–3 days.

For western blotting, Caco-2 cell monolayers were grown on 6-well plates at a density of 4.0 × 10^5^ cells/well. The cell monolayers were maintained for 18–21 days and culture medium was replaced every 2–3 days.

For globotriaosylceramide (Gb3) suppression, Caco-2 cell monolayers were grown on Transwell inserts in 24-well plates at a density of 1.0 × 10^5^ cells/cm^2^. The apical and basolateral compartments contained 0.1 and 0.6 mL of culture medium with 0.2 µM or 2.0 µM PPMP, respectively. The cell monolayers were maintained for 18–21 days and culture medium was replaced every 2–3 days. The integrity of the cell monolayers was evaluated by measuring TER values. The cell monolayers showing TER values of more than 500 Ω/cm^2^ were used for the experiments.

### 3.3. Effect of Lectins on Tight Junction (TJ)

Caco-2 cells seeded in Transwell inserts were treated with lectins (20, 100, and 200 μg/mL) for 2–6 h. The TER values of the cell monolayers were measured to investigate the effect of lectins on the TJ. The TER values were also measured at 2 h after the addition of 10 mM saccharides to apical solution.

### 3.4. LY Transport Experiment

The cell monolayers on Transwell inserts were gently rinsed twice with pre-warmed HBSS and incubated HBSS for 30 min at 37 °C. After incubation, TER values were measured to confirm the differentiation of Caco-2 cells. After removing the apical and basolateral solution, to apical sides were added HBSS containing CSL3 (100 μg/mL) with 10 µM of LY and to basolateral sides were added HBSS. After incubation, TER values were measured again. The basolateral solutions were taken and subjected to reverse-phase HPLC analysis on a Capcelpack ODS AG120 column (4.6 × 150 mm) (Shiseido, Tokyo, Japan) at a flow rate of 1.0 mL/min at 40 °C. Solvent A was 25 mM sodium acetate (pH 7.0), and solvent B was 25 mM sodium acetate (pH 7.0)–acetone (50:50, *v*/*v*). A linear gradient elution was carried out from 0 to 100% solvent B in 15 min. Detection was with a fluorescence detector at excitation and emission wavelengths of 450 and 540 nm, respectively.

### 3.5. Intracellular Ca^2+^ Measurement

The cells were incubated with CSL3 (20, 100, 200 μg/mL) in HBSS for 2 h at 37 °C, and then with 5 µM Fluo4-AM, 0.025% pluoronic F-127, and 2 mM probenecid in HEPES-buffer (20 mM HEPES, 115 mM NaCl, 5.4 mM KCl, MgCl_2_, 1.8 mM CaCl_2_, and 13.8 mM glucose , pH 7.4) for 1 h at 37 °C. After removing the solution, the fluorescence of the cells in HEPES-buffer was measured with a fluorescence detector at an excitation and emission wavelengths of 485 and 530 nm, respectively. The fluorescence of the cells was also measured when the cells were incubated in the presence of 10 mM saccharides for 1 h after the lectin treatment.

### 3.6. Fluorescence Immunostaining

The cells were incubated with CSL3 (100 µg/mL) in HBSS for 2 h at 37 °C. The cells were fixed in 4% paraformaldehyde in phosphate buffered-saline (PBS). Following permeabilization in 0.2% Triton X-100 in PBS, the cell monolayers were blocked in 1% skim milk in PBS. After a 1-h incubation at room temperature, the cell monolayers were incubated with primary antibody (rabbit polyclonal anti-β-actin) at 4 °C overnight, followed by secondary antibody (Alexa Fluor 488 conjugated goat anti-rabbit IgG antibody) for 1 h. After staining with 4',6-diamidino-2-phenylindole (DAPI) for 20 min, the fluorescence images were examined by an epifluorescence microscope, and stacked using metamorph software. The fluorescence images were also examined when the cells were incubated in the presence of 10 mM l-rhamnose for 1 h after CSL3 treatment.

### 3.7. Western Blotting

The cell monolayers were gently rinsed twice with HBSS, and treated with CSL3 (200 µg/mL) for 2 h. The cells were washed with cold PBS and lysed in cold buffer (containing 50 mM Tris-HCl, pH 7.4, 150 mM NaCl, 1% Triton X-100, 1 mM EDTA, 0.25% sodium deoxycholate) with 1 mM NaF, 10 µM Na_3_VO_4_, 1 mM phenylmethanesulfonyl fluoride (PMSF), and a protease inhibitor cocktail (10 µg/mL leupeptin, 10 µg/mL aprotinin, and 1 µM pepstain), at 4 °C for 1 h. After centrifugation at 12,000 rpm for 10 min at 4 °C, protein concentrations were determined by the bicinchoninic acid (BCA) protein assay. Subsequently, 40 µg of proteins (for claudin-1, β-actin, glyceraldehyde-3-phosphate dehydrogenase (GAPDH) were denatured in SDS-PAGE sample buffer. The proteins were separated by SDS–PAGE (15% gel) and transferred onto PVDF membrane (Millipore) by electrobolotting for 1 h under semidry conditions with a Transblot SD apparatus (Bio-Rad Laboratories, Hercules, CA, USA). Non-specific binding was blocked with blocking buffer (1% skim milk and 0.1% Tween-20 in PBS) for 1 h at 37 °C. The membranes were incubated with specific antibodies against claudin-1 or GAPDH (1/1000 dilution) in blocking buffer overnight at 4 °C. After washing three times with PBS containing 0.5% Tween-20, the membranes were incubated with HRP-conjugated goat anti-rabbit IgG (1/5,000 dilution) in blocking buffer. The blots were developed with Luminata Forte Western HRP substrate (ATTO, Tokyo, Japan) and observed by a CS Analyzer (ATTO).

### 3.8. Data Analysis

The results are shown as the percentages of control values, and expressed as the mean ± SD of three or six individual determinations. Significant difference was analyzed by the Student’s *t*-test, and differences with *p* < 0.05 were considered to be significant.

## 4. Conclusions

A chum salmon egg lectin (CSL3) opened the tight junction (TJ) of Caco-2 cell monolayers to decrease the transepithelial electrical resistance (TER) value and increase of the permeation of lucifer yellow by interacting with sugar chains on the cell surface. The decreased TER values were increased within an hour by the addition of specific sugars, l-rhamnose or d-galactose. The effect of the lectin may be related to increase of the intracellular Ca^2+^ followed by depolymerization of β-actin without affecting the expression of TJ proteins such as claudin-1.
